# Training in Temporal Information Processing Ameliorates Phonetic Identification

**DOI:** 10.3389/fnhum.2018.00213

**Published:** 2018-06-06

**Authors:** Aneta Szymaszek, Anna Dacewicz, Paulina Urban, Elzbieta Szelag

**Affiliations:** Laboratory of Neuropsychology, Nencki Institute of Experimental Biology of Polish Academy of Sciences, Warsaw, Poland

**Keywords:** temporal information processing (TIP), specific language impairment (SLI), voice-onset-time, phonetic identification, temporal intervention, voicing contrast detection

## Abstract

Many studies revealed a link between temporal information processing (TIP) in a millisecond range and speech perception. Previous studies indicated a dysfunction in TIP accompanied by deficient phonemic hearing in children with specific language impairment (SLI). In this study we concentrate in SLI on phonetic identification, using the voice-onset-time (VOT) phenomenon in which TIP is built-in. VOT is crucial for speech perception, as stop consonants (like /t/ vs. /d/) may be distinguished by an acoustic difference in time between the onsets of the consonant (stop release burst) and the following vibration of vocal folds (voicing). In healthy subjects two categories (voiced and unvoiced) are determined using VOT task. The present study aimed at verifying whether children with SLI indicate a similar pattern of phonetic identification as their healthy peers and whether the intervention based on TIP results in improved performance on the VOT task. Children aged from 5 to 8 years (*n* = 47) were assigned into two groups: normal children without any language disability (NC, *n* = 20), and children with SLI (*n* = 27). In the latter group participants were randomly classified into two treatment subgroups, i.e., experimental temporal training (EG, *n* = 14) and control non-temporal training (CG, *n* = 13). The analyzed indicators of phonetic identification were: (1) the boundary location (α) determined as the VOT value corresponding to 50% voicing/unvoicing distinctions; (2) ranges of voiced/unvoiced categories; (3) the slope of identification curve (β) reflecting the identification correctness; (4) percent of voiced distinctions within the applied VOT spectrum. The results indicated similar α values and similar ranges of voiced/unvoiced categories between SLI and NC. However, β in SLI was significantly higher than that in NC. After the intervention, the significant improvement of β was observed only in EG. They achieved the level of performance comparable to that observed in NC. The training-related improvement in CG was non-significant. Furthermore, only in EG the β values in post-test correlated with measures of TIP as well as with phonemic hearing obtained in our previous studies. These findings provide another evidence that TIP is omnipresent in language communication and reflected not only in phonemic hearing but also in phonetic identification.

## Introduction

### Characteristics and Associated Features of Specific Language Impairment

Specific Language Impairment (SLI, diagnosed as F.80.1 and F.80.2 according to ICD 10; Pużyński and Wciórka, 2000) is a form of developmental language impairment in which children demonstrate difficulties in understanding and/or producing speech. However, their general cognitive functioning and non-verbal intelligence remain within the normal range. The language impairment cannot be explained by hearing problems, neurological and speech mechanism abnormalities or environmental factors. The prevalence of SLI is estimated to be approximately 7% among 5-year-olds (Tomblin et al., 1997).

Specific language impairment generates pervasive social problems possibly in relations to future lower academic achievements. Hence, there is a necessity to identify the causal factors of SLI and to create the efficient speech therapy which may provide language disordered children with the same opportunities as their typically developing peers.

Although developmental language disorders have been investigated for almost 150 years, the neural basis of SLI still remains unclear. One theoretical approach assumes difficulties associated with deficient perception of auditory input. In the early seventies Tallal and colleagues revealed that SLI children are less efficient in discriminating between verbal (Tallal and Piercy, 1974; Tallal, 1975) and non-verbal (Tallal and Piercy, 1973; Tallal, 1975) sounds presented in rapid succession. Since that time, the researchers considered difficulties in temporal information processing (TIP) of rapidly changing acoustic events as one of the core problems in SLI.

This indication is in line with a long discussion about the relationship between TIP and language in norm and pathology. Several subject populations, including children with language-learning-impairment (Benasich and Tallal, 2002; Fitch and Tallal, 2003), children or adults with dyslexia (Tallal et al., 1995; Rey et al., 2002) and patients with aphasia following left hemisphere brain lesions (Swisher and Hirsh, 1972; von Steinbüchel et al., 1999; Wittmann et al., 2004; Fink et al., 2006) displayed deficits in the perception of temporal order of two stimuli presented in rapid succession. They indicated elevated temporal order threshold (TOT), i.e., they needed longer time interval between two sounds to report correctly their temporal relation ‘*before-after.*’

In our previous study (Szelag et al., 2015) the coexistence of deficient TIP and disordered phonemic hearing was confirmed *inter alia* in children with SLI. We indicated that they displayed higher TOT (of about 184 ms) than normal peers (about 96 ms) accompanied by deficient phonemic hearing. In the present study we verify in children with SLI the co-occurrence of these deficits with disordered phonetic identification using the voice-onset-time (VOT) which may be considered as a sensitive measure combining TIP with phonetic aspects of speech perception.

### TIP in Voiced/Voiceless Categorization

Speech perception requires neural encoding of both spectral acoustic and temporal cues. Different speech sounds (consonants and vowels) vary in their spectrotemporal characteristics. Whereas vowels present a relatively steady-state pattern of formants, stop consonants are temporally much more transient and acoustically variable (Obleser et al., 2007). Subjects with deficient TIP have accompanying difficulties in stop consonants reception which are critical in time, whereas such deficits rarely affect the perception of vowels. Moreover, stop consonant – vowel syllables (e.g., /TO/ and /DO/) are distinguished by acoustic differences in time between the onset of the consonant (stop release burst) and the onset of the following vowel (voicing). Such voiced/unvoiced categorical perception is the most frequently studied phonetic feature. It is measured with the VOT phenomenon defined as the time interval between the release from stop closure (onset of the consonant) and the onset of vibration of the vocal folds (onset of the following vowel, Lisker and Abramson, 1964; Molfese, 1980). It is worth mentioning that different languages are characterized by various temporal cues for VOT, thus, the relationship between the burst and laryngeal pulsing. The positive VOT reflects a situation when the laryngeal pulsing is preceded by a burst. In contrast, in case of negative VOT the burst is preceded by the laryngeal pulsing (Szelag and Szymaszek, 2014).

Some languages like American English or German use only the positive VOT, reflected in values from 0^1^ to 20 ms for voiced categorizations, like /BA/, /DA/, /GA/ and longer intervals of around 30–80 ms for voiceless categorizations, like /PA/, /TA/, /KA/ (King et al., 2008). In other languages, e.g., Slavic or French the negative VOT is also observed. The voiced contrast detection varies along a continuum of VOT values. Among Slavic-speaking (including Polish) environments these values are located from –90 to 20 ms. Thus, the differentiation between voiced/unvoiced contrast bases on both negative and positive VOT.

Independently of these cross-linguistic differences in voiced/unvoiced categorical perception some language *universalia* emerge. They comprise a similar time gap of some tens of milliseconds with either positive or negative values critical for such differentiation in a given language. The efficient TIP in the millisecond time range seems to be crucial for the categorical voiced/unvoiced contrast perception, independently of the natural language. VOT phenomenon as an important aspect of phonetic identification in speech perception has been a topic of many studies including both normal and clinical populations (Giraud et al., 2007; King et al., 2008; Doellinger et al., 2011; Chobert et al., 2012).

### Timing-Related Approaches to Remediation of Language Acquisition

As difficulties in rapid auditory processing were indicated as the crucial deficit at least in some children with SLI and the conventional speech therapy often seems not efficient enough, the interventions based on TIP were developed (Merzenich et al., 1996; Tallal et al., 1996; Wittmann and Fink, 2004; Cohen et al., 2005; Gillam et al., 2008; Given et al., 2008). The widely known remediation software Fast ForWord^®^ was successfully implemented in children with SLI and resulted in improvement of expressive and receptive language skills. In addition, the effectiveness of the *Dr. Neuronowski^®^* software, focused on TIP, developed in our Institute (Szelag and Szymaszek, 2016), was verified in children with SLI in our previous study (Szelag et al., 2015). We found that such intervention resulted in lowered TOT values, reflecting improved TIP in comparison to the non-significant change after control non-temporal training. The improvement in TIP was accompanied by amelioration of language skills in both phonemic hearing and global speech comprehension tasks.

### Study Aims

In the present study we tested whether children with SLI present the same boundaries for the typical categories of phonetic identification as their healthy peers and whether the application of intervention based on rapid auditory processing may result in improved performance on VOT task.

## Materials and Methods

### Participants

Forty-seven right-handed (Edinburgh Inventory) children aged between 5 and 8 years participated in the study. They were classified into two groups: (1) normal children without any language disability (NC, *n* = 20) and (2) children affected by SLI (*n* = 27). In the latter group children were randomly assigned using the RITA^®^ software (Pahlke et al., 2004) into two intervention subgroups, i.e., experimental temporal training subgroup (EG, *n* = 14) and control non-temporal training subgroup (CG, *n* = 13).

All children were monolingual Polish native speakers. NC were recruited at kindergartens in the area of Warsaw, whereas children with SLI at either the Early Intervention Centre or the Children’s Memorial Health Institute in Warsaw. All participants showed normal hearing level (ANSI, 2004) which was verified with screening audiometry for 500, 1000, 2000, and 4000 Hz frequencies (audiometer AS 208). These frequencies covered the frequency spectrum of auditory stimuli presented in this study. All children had normal level of non-verbal intelligence (IQ at least 85 or higher, measured by the Raven’s Colored Progressive Matrices; Szustrowa and Jaworowska, 2003).

In case of children with SLI the developmental language impairment was defined as reduced language competency, evidenced by the Test for Assessment of Global Language Skills (TAGLS; Tarkowski, 2001). It constitutes the screening assessment tool for language development in Polish children. All participants with SLI obtained the overall standard language score on at least two standard language subtests below or equal 4th sten. The exclusion criteria were neurological and psychiatric disorders, attention deficits or socio-emotional disorders (as determined by the parental report) and the participation in any other therapy program during our data collection which might have influenced the obtained results.

All three groups (NC, EG, and CG) were balanced according to age, gender, non-verbal IQ based on either one-way analysis of variance (ANOVAs) (for age and IQ) or Pearson’s chi-squared test (for gender). Controlling such variables was important for the efficacy of the applied interventions. It was a blinded randomized controlled study. The detailed subject characteristics are given in **Table 1**.

**Table 1:** The detailed characteristic of the subject pool.

	Children without any language disability (NC)	Children with specific language impairment (SLI)	
		Experimental temporal training subgroup (EG)	Control non-temporal training subgroup (CG)
Number of subjects	20	14	13
Age (mean ± SD, years)	6.2 ± 0.8	6.4 ± 0.9	6.0 ± 0.9
Gender (boys/girls)	13/7	9/5	10/3
IQ (mean ± SD)	120 ± 14	110 ± 12	114 ± 16

### Ethics Statement

The study protocol was approved by the Bioethical Commission at the Warsaw Medical University (Permission No. KB/162/2010). Prior to testing a written informed consent was obtained from the parents of each child participating in the study; children provided a verbal approval.

### Stimuli and Experimental Procedures

The study comprised both assessment and intervention procedures. The assessment procedures included three tasks: (1) phonetic identification tested with VOT Task (Szelag and Szymaszek, 2014), (2) phonemic hearing with Phoneme Discrimination Test (PDT, Szelag et al., 2015), and (3) TIP with auditory TOT (Fink et al., 2006). These assessment procedures in SLI group were performed twice, i.e., before (pre-test) and, next, after (post-test) the applied intervention. In case of NC the phonetic identification data were collected at the beginning of the study, this group reminded without any intervention.

#### Assessment Procedures

##### Measurement of phonetic identification

Voice-onset-time task is a sensitive tool for evaluation of phonological deficits on a basis of phonetic identification during speech perception. The task has built-in the millisecond TIP component which is crucial for a differentiation between *voiced* and *unvoiced* category at the initial bilabial consonant. It was achieved by parametrizing a single acoustic temporal dimension of VOT across the synthetized spectrum of presented stimuli. The stimulus continuum was selected on a basis of our previous studies (Szelag and Szymaszek, 2014).

**FIGURE 1:**
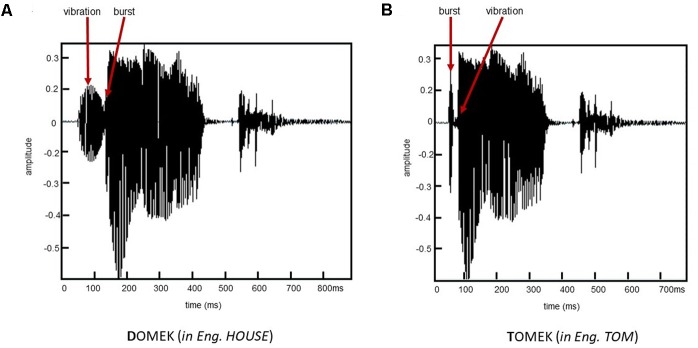
Waveforms of two words from the endpoints of applied VOT spectrum: **(A)** VOT of –90 ms identified as **D**OMEK and **(B)** Voice-onset-time (VOT) of 20 ms identified as **T**OMEK.

A series of voiced/unvoiced stimuli were created on a basis of the Polish word /**T**OMEK/ (with *unvoiced* initial consonant, naturally spoken with a female voice, *in English:* Tom). In all created stimuli the segment /OMEK/ was spectrally identical. The voiced/unvoiced contrast was achieved by the manipulation (Adobe Audition 3.0) in a single acoustic dimension of VOT within the initial consonant in semi-synthesized word **T**OMEK. The created stimuli differed in VOT values that separated the onset of the stop burst and subsequent voicing, thus, the relationship between the burst and laryngeal pulsing. It created a continuum of VOT values comprising 13 stimuli: –90, –80, –70, –60, –50, –40, –30, –20, –10, 0, +5, +10, and +20 ms. For VOT from –90 to –10 ms the burst was preceded by the laryngeal pulsing (negative VOT), while from +5 to +20 ms the laryngeal pulsing was preceded by a burst (the positive VOT; for explanations of positive vs. negative VOTs see section “Introduction”). Accordingly, in Slavic languages (including Polish) the word of –90 ms VOT is identified as **D**OMEK, whereas, that of 20 ms as **T**OMEK with transition zone (chance level identification) for VOTs of –30 and –20 ms. Illustrative waveforms of two endpoint stimuli of applied VOT continuum are shown in **Figure 1**.

**FIGURE 2:**
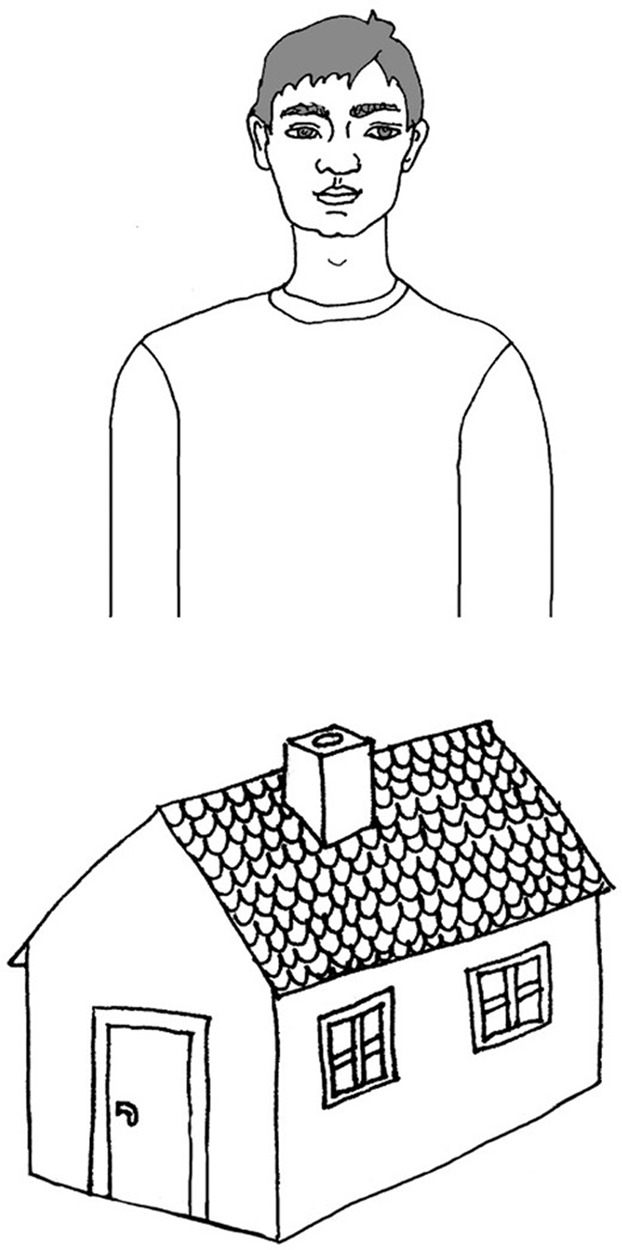
The response card used in VOT test.

Children were presented with these stimuli binaurally through headphones at a comfortable listening level. The measurement based on identification of presented words as either **/**TOMEK**/** or **/**DOMEK**/**. Children were asked to listen to the presented words and to associate each stimulus they heard with one of two pictures. These pictures were presented on one response card (format A4, **Figure 2**). The upper picture displayed a boy named Tom (in Polish: **/T**OMEK/) and the lower picture a house (in Polish: **/D**OMEK**/**).

In each child the experiment was preceded with an introductory practice session. First, the children were introduced to the above two pictures, hearing examples of **/D**OMEK**/** and **/T**OMEK**/** with the VOT corresponding to continuum endpoints (-90 and 20 ms). Next, 16 presentations (8 repetitions of 2 words from the VOT continuum endpoints, i.e., –90 ms and 20 ms) were randomly presented. After each presentation children were requested to point to the proper picture and a feedback on the correctness achieved was given. The introductory practice session ended when four responses in a row were correct. Then, the experiment started without any feedback on the correctness achieved.

The measurement comprised 78 presentations (6 series, each consisted of the 13 basic stimuli presented in random order).

###### Outcome measures

We analyzed the percent of voiced distinctions within the whole VOT spectrum.

In further analyses the psychometric (sigmoid) function^2^ was adjusted to all responses given by each child (Treutwein and Strasburger, 1999; Strasburger, 2001). This function based on the percent of voiced /DOMEK/ identifications for each of the 13 applied VOT values. The sigmoid function was the Z-shape curve limited in the range from 0 to 1 values. The 0 value for a defined VOT stimulus corresponded to a lack of any voiced identification in child’s responses, whereas the value 1 meant 100% of such detections. The sigmoid function (*f*(*x*)) was defined by the formula:

$$f(x)=\frac{1}{1+\exp\;(\frac{x-a}{\beta })}$$

where α reflects the categorical boundary location (*x* coordinate of ‘half way up”) corresponding to 50% voiced/unvoiced discriminations and β indicates the slope of the identification curve. In such generalized logistic model differences in categorical precision are reflected by a shallower slope of the function, i.e., the higher β the more flat curve (less categorical perception) corresponding to the worse performance. The additional abbreviations in the above formula are:

• *x –* particular VOT values (from –90 to +20 ms) reflecting the spectrum applied here;
• exp – exponential function, i.e., a logarithmic function based on a natural logarithm *e* = 2.78 (the Euler’s number);

In our data analysis both α and β values were used as indicators of phonetic identification (see section “Results”).

##### Measurements of phonemic hearing and TIP

As stated before, the efficiency of phonemic hearing was evaluated with the PDT, whereas that of TIP with auditory TOT task. The measurement procedures of PDT and TOT were described in detail in our earlier report (Szelag et al., 2015). In the present study we refer to some data collected previously and published in Szelag et al. (2015; see Tables 2, 3, p. 9). From the subject pool published in this previous report (*n* = 32) we selected the data of 27 children with SLI (considering EG and CG subgroups) who were tested also with the VOT task in the present study. These previous data were used here to test in EG and CG the correlations between phonemic hearing (or TIP) and β value used as an indicator of phonetic identification in VOT task performance. Below we summarize briefly the measurement procedures of phonemic hearing and TIP.

Phoneme Discrimination Test comprises 64 paired-words in which 75% pairs were different, e.g., górnik – kurnik (*in English:* miner – hen house) and 25% the same, e.g., mama –mama (*in English:* mother – mother). The task was to judge whether two words within the presented pair were the same or different. Responses were given by pointing to one of the two response cards, corresponding to these two situations. In case of different paired-words they did not match in one phoneme. The differed paired-words contrasted for place of articulation, plosive, fricative, voicing, or nasality.

In TIP the measurement based on auditory TOT defined as the minimum time gap between two auditory stimuli presented in rapid succession that is necessary for a participant to report correctly their temporal order, i.e., the relation *before-after* at 75% correctness. The stimuli were paired 1 ms clicks presented monaurally (i.e., to each ear separately) with various inter-stimulus-intervals (ISIs). The task was to report the order of two clicks, thus: *left–right* or *right–left*. ISIs varied adaptively from 1 to 600 ms, according to the adaptive maximum-likelihood-based algorithm (Treutwein, 1997) until the TOT was located with a probability of 95% inside a ±5 ms interval around the currently estimated threshold (Treutwein, 1995).

#### Intervention Procedures

As mentioned above, in children with SLI two types of interventions were applied, i.e., experimental temporal intervention (in EG subgroup) and control non-temporal intervention (in CG). Detailed description of both intervention programs was provided in Szelag et al. (2015).

##### Experimental temporal intervention

Experimental temporal intervention procedure used the multimedia intervention program *Dr. Neuronowski.^®^* It was designed in our Institute on the basis of our previous prototyping interventions addressed TIP (Szelag et al., 2014; Szymaszek et al., 2017). This software consists of nine various modules containing 46 basic computer games. The crucial aspect of this software is that the majority of games involved TIP in the millisecond time range, sequencing abilities or duration judgment. Moreover, the temporal-based games were extended by tasks exercising other cognitive functions, i.e., language comprehension, attention, working memory and executive functions. The software was auto adaptive, i.e., task difficulty changed adaptively based on correctness of the actual child’s performance. The tasks difficulty was modified according to numerous parameters, i.e., number, length and presentation rate of verbal and non-verbal stimuli, rate of modified speech, various ISIs in stimuli presented sequentially, application of various distractors, time limitations for responses.

##### Non-temporal control intervention

Non-temporal control intervention was based on freely available computer games (e.g., *Memory or Tetris*), as well as on educational speech-therapy exercises. The combination of such software trained phonemic hearing, articulation and vocabulary, as well as attention, working memory and executive functions. Contrary to the experimental temporal intervention, none of these tasks engaged any rapid auditory processing in the millisecond time range.

#### Study Protocol

**FIGURE 3:**
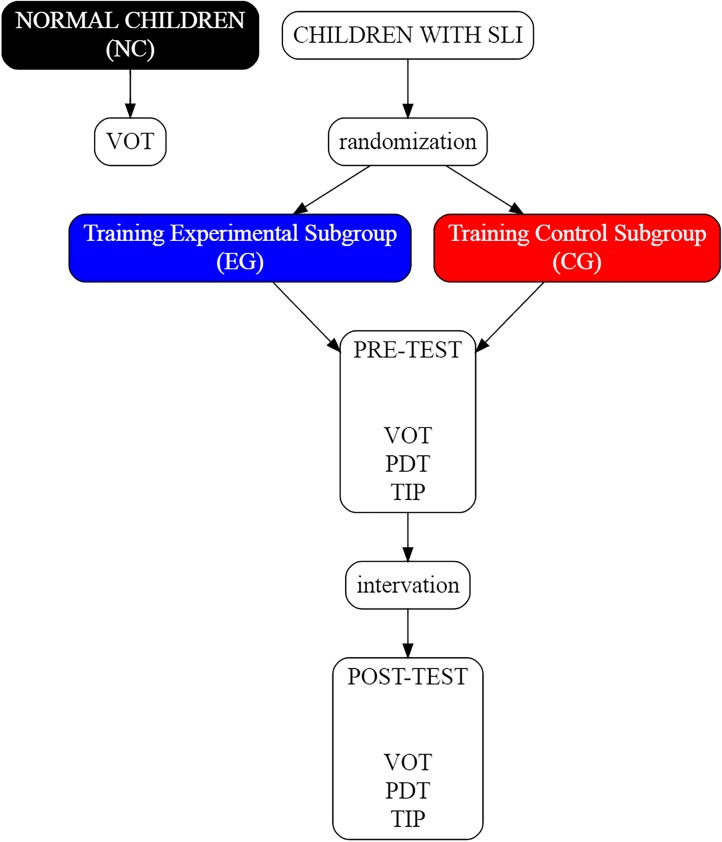
Schema of the study. In NC the VOT task was performed once, whereas in EG and CG subgroups all three assessment tasks: VOT, Phoneme Discrimination Test (PDT), and temporal information processing (TIP) were conducted twice, i.e., before and after the intervention.

The assessment procedures and the intervention programs (**Figure 3**) were conducted with each child individually. The intervention (experimental and control) consisted of 24 sessions of 1-h each, performed 3 times per week.

### Statistical Analyses

**FIGURE 4:**
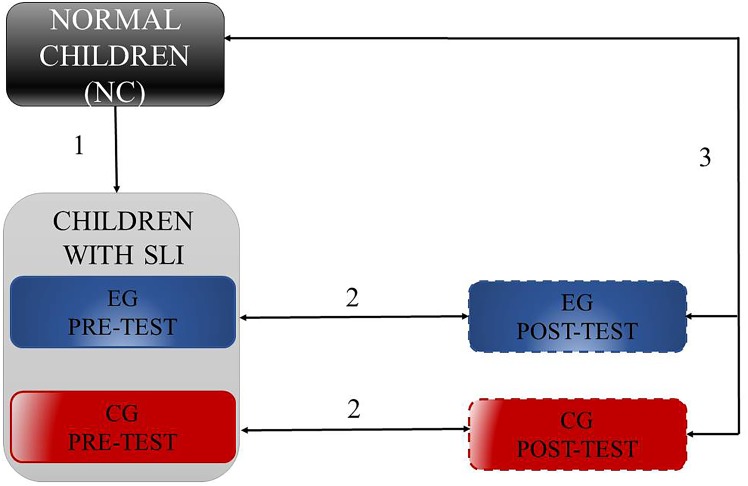
Schema of performed comparisions in Steps 1–3, where (1) reflects camparisons of phonetic identification between NC and SLI, (2) training-related differences in EG and CG, and (3) post-test performance in EG and CG in comparison to NC.

Statistical analyses comprised four Steps. They included: (1) comparison of phonetic identification between NC and SLI children, (2) training-related differences separately in EG and CG, (3) post-test performance in EG and CG in comparison to NC, and (4) relationships in EG and CG between the phonetic identification indexed with the function slope (β) and results of phonemic hearing and TIP in pre- and in post-test assessment, separately. In Steps 1–3 (**Figure 4**) we analyzed both α and β^3^ values using the *U* Mann–Whitney test (Step 1), Wilcoxon Signed-Rank test (Step 2) and Kruskal–Wallis one-way ANOVA followed by the *U* Mann–Whitney tests (Step 3).

Additionally, in Step 1 these comparisons were extended by 2-way ANOVA with the percent of voiced responses for the whole spectrum of VOT values in NC and SLI. This ANOVA aimed at testing the differences in phonetic identification for particular VOT stimuli within the voiced, unvoiced and transition categories^4^. It included ‘Group’ (NC vs. SLI) as between-subject factor and ‘VOT value’ (13 values: –90, –80, –70, –60, –50, –40, –30, –20, –10, 0, +5, +10, and +20 ms) as a within-subject factor^5^. In SLI group only pre-test (summed EG and CG) data were considered.

In Step, 4 using Spearman correlations, we tested the relationships between phonetic identification indexed with β values and other cognitive skills reflected by phonemic hearing and TIP in EG and CG subgroups in pre- and in post-test assessment, separately. Referring to Szelag et al. (2015), TOT was the indicator of TIP efficiency and PDT of phonemic hearing.

## Results

### Phonetic Identification in NC and SLI

**FIGURE 5:**
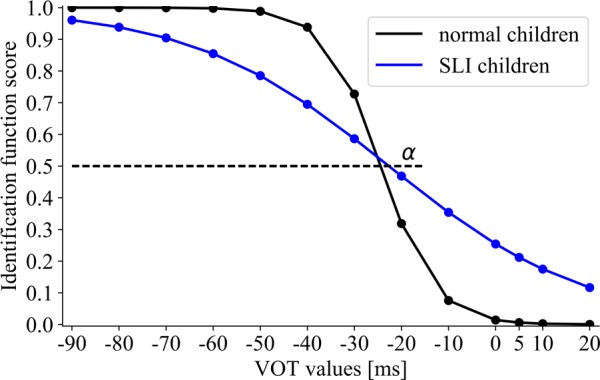
The sigmoid function for NC and specific language impairment (SLI) groups. The boundary location (α) corresponds to the VOT value at which 50% of voiced/unvoiced identifications were detected.

Between-group differences for *the boundary location* (assessed with α, **Figure 5**) were non-significant (*U* = 244, *p* = 0.59). The obtained results indicated the similar boundaries for voiced/unvoiced distinctions in NC (α = –24.5) and SLI (α = –22.2).

Significant between-group differences for *the slope of identification function* (assessed with β, **Figure 5**) were found (*U* = 96, *p* = 0.0002). The NC group presented the steeper slope (β = 3.62) than the SLI group (β = 26.3), corresponding to better performance in the former group.

**FIGURE 6:**
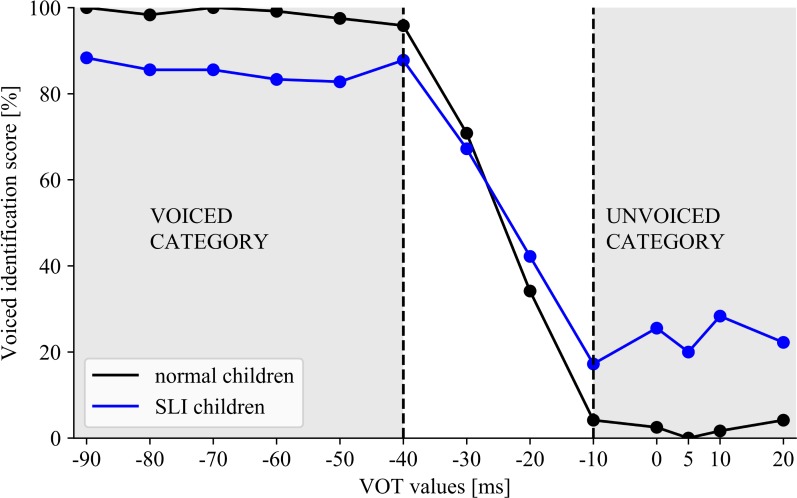
The voiced identification score for the presented VOT spectrum in NC and SLI indicating the similar ranges of voiced/unvoiced categories in both groups with poorer performance in SLI than in NC.

Analysis of variance with the voiced identification scores for the VOT spectrum revealed a significant effect of ‘VOT value’ [*F*(12/540) = 271.593, *p* < 0.001, η^2^ = 0.858] modified by the interaction ‘VOT value’ × ‘Group’ [*F*(12/540) = 9.313, *p* < 0.001, η^2^ = 0.171]. In both groups the results were patterned by two phonetic categories (**Figure 6**). The voiced category comprised the VOT values ranged from –90 to –40 ms, whereas, the unvoiced one from –10 to 20 ms, independently of the group. The ranges of these two categories were established on a basis of significant jump in the identification score between –40 and –30 ms (*p* < 0.001 in both groups) indicating the voiced category and between –20 and –10 ms (*p* < 0.001 in both groups) for the unvoiced category. Despite these similarities in both groups, the better performance within each category was observed in NC than in SLI (‘VOT value’ × ‘Group’ interaction).

### The Effect of Applied Experimental vs. Control Intervention

**FIGURE 7:**
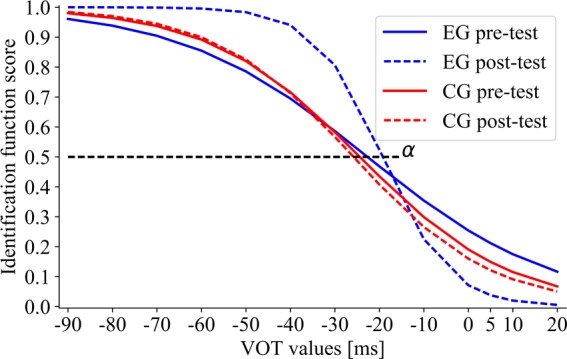
The sigmoid function for EG and CG subgroups in pre- and post-test performance. The boundary location (α) corresponds to the VOT value at which 50% of voiced/unvoiced identifications were detected.

The effect of training on *the boundary location* (**Figure 7**) was non-significant in EG (*Z* = 1.10; *p* = 0.27; α_pre-test_ = –21.80 and α_post-test_ = –19.93) and in CG (*Z* = 0.73; *p* = 0.47; α_pre-test_ = –22.63 and α_post-test_ = –24.34). Thus, the categorical boundary location remained relatively stable following each type of intervention.

The training-related changes in *the slope of identification function* indicated significantly lower β in EG in post- than in pre-test (*Z* = 2.73; *p* = 0.007; β_pre-test_ = 29.59, β_post-test_ = 6.72), corresponding to improved performance (**Figure 7**). In contrast, β in CG did not differ significantly between post- and pre-test (*Z* = 0.52; *p* = 0.60; β_pre-test_ = 22.77, β_post-test_ = 19.80), indicating the similar level of performance in pre- and post-test.

### Post-Test Performance in EG and CG in Comparison to NC

**FIGURE 8:**
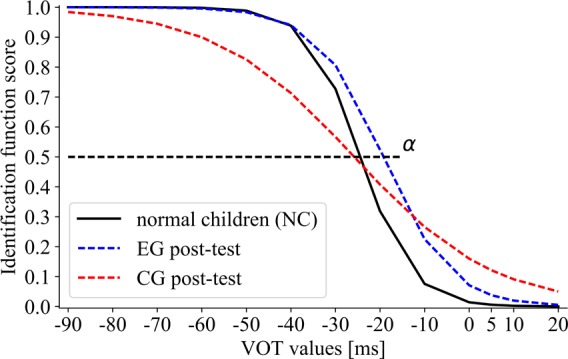
The sigmoid function for post-test performance of EG and CG subgroups in comparison to NC. The boundary location (α) corresponds to the VOT value at which 50% of voiced/unvoiced identifications were detected.

Between-group comparisons for *the boundary location* (**Figure 8**) were non-significant (*H* = 3.5, *p* = 0.18; α_EG_ = –19.93, α_CG_ = –24.34, α_NC_ = –24.54).

Significant between-group differences in *the slope of identification function* were observed (*H* = 7.61, *p* = 0.03) between CG and NC (*U* = 62, *p* = 0.02; β_CG_ = 19.80, β_NC_ = 3.62). On the contrary, differences between EG and NC were non-significant (*U* = 139, *p* = 0.99; β_EG_ = 6.72, β_NC_ = 3.62). The obtained results indicated that EG in post-test reached the level of NC performance, whereas the post-test performance in CG remained still significantly below that of NC.

### Relationships Between Phonetic Identification and Other Cognitive Skills

In pre-test the β values did not correlate significantly with TOT (*r* = 0.23, *p* = 0.43 for EG and *r* = –0.09, *p* = 0.78 for CG) and PDT (*r* = 0.48, *p* = 0.09 for EG and *r* = 0.22, *p* = 0.47 for CG) in any subgroup. In contrast, in post-test only in EG significant correlations were found between β and TOT (*r* = 0.58, *p* = 0.03), as well as between β and PDT scores (*r* = 0.56, *p* = 0.04). For CG correlations between β and TOT (*r* = 0.25, *p* = 0.42), as well as between β and PDT scores (*r* = 0.15, *p* = 0.62) were non-significant.

## Discussion

Considering the aims of our study, the discussion of obtained results is focussed, firstly, on the differences in phonetic identification between children with SLI and healthy peers, followed by the training-related changes in such identification. Finally, we concentrate on relationships between the level of phonetic identification assessed with the VOT test and results of TIP and phonemic hearing obtained in our previous study (Szelag et al., 2015).

### Voicing Contrast Detection in Children With SLI on the Background of Healthy Controls

Although some existing literature studies concern the phonetic identification in children with SLI, the boundaries of categorical perception of voiced/unvoiced detection in such children have been rarely studied. Therefore, the important result of the present study was the indication of non-significant differences between the boundary location (α) in children with SLI and NC (**Figure 5**). Moreover, in both these groups the similar ranges of categories for voiced/unvoiced detection were distinguished. The voiced category was identified at VOT values from –90 to –40 ms, while the unvoiced category from –10 to 20 ms (ANOVA, **Figure 6**) which is congruent with the previous reports in Polish subjects (Rojczyk, 2010). Besides these similarities, deficient voiced contrast detection reported in this study was reflected in significantly lower correctness within these categories in children with SLI than in NC (**Figures 5**, **6**). It was evidenced in statistical analyses (ANOVA) as well as in a shallower slope of identification function (higher β) values in children with SLI which corresponds to worse performance.

As mentioned above, speech perception difficulties have been frequently reported in SLI in literature studies. For example, Ziegler et al. (2005, 2011) investigated the perception of speech in noise considering various features, such as: voicing, manner and place of articulation. Although the perception of all these features was impaired in children with SLI, as compared to age-matched healthy controls, the voicing was impaired to a greater extent. It was interpreted as the strongest deficit compared to other features of speech perception. The deficient voiced categorical perception may reflect the deteriorated millisecond TIP which is incorporated in such voiced/unvoiced categorization (Benasich and Tallal, 2002; Fitch and Tallal, 2003; Szelag et al., 2015).

As indicated before, particular languages are characterized by specific boundaries for voiced detection (see section “Introduction”). Nevertheless, literature studies provided evidence that infants who experienced any linguistic environment are sensitive to some universal boundaries of phonetic identification which were located at VOT values between –30 and 30 ms (Lasky et al., 1975; Streeter, 1976). It was concluded that infants have a specialized biological predisposition to discriminate an universal set of phonetic contrasts (Eimas, 1991). The process of language acquisition during child development involves reorganization of this universal sensitivity under the influence of specific environmental conditions. Based on this view, infants at around 6 months of life transfer from a language-general to a language-specific mode of speech perception with phonetic boundaries typical for the experienced language. It was evidenced for Spanish (Lasky et al., 1975) as well as for French (Hoonhorst et al., 2009). One may expect that the skilled functioning in the range of some tens of milliseconds is crucial for language development from early years of life. In the VOT task, similarly as in the TIP task, it is necessary to perceive effectively two sequential events (e.g., burst–vibration or two sounds) separated in time by some tens of milliseconds. Such a statement is supported by the literature evidence on shared neural network for rapid auditory processing and speech processing (e.g., Zaehle et al., 2004).

A number of studies has revealed deficient categorical contrast detection in children with SLI. For example, Sussman (1993) compared the performance of VOT in syllables, using the discrimination and identification methods. During discrimination measurement the performance in children with SLI was comparable to that reported in healthy controls. In contrast, in identification they were significantly less accurate. The impaired phonetic identification in VOT continuum in children with SLI was further confirmed by Gerrits and de Bree (2009). Our results are congruent with these literature reports (**Figures 5**, **6**). Although several studies indicated lower accuracy of phonetic identification in children with SLI, our important finding was the observation that categorical boundaries for voiced/unvoiced identification were still preserved and remained comparable to those observed in healthy controls. At this point what should be noted is the similar boundary location reflected in α (**Figure 5**) as well as typical voiced/unvoiced category ranges (**Figure 6**) in 5–8-year-old normally developing children and in SLI ones. The indication of the typical temporal framework for phonetic identification in SLI seems to be promising in the context of speech neurorehabilitation.

### Training-Related Changes in Voiced Contrast Detection

Despite existing literature controversies on the contribution of deficient TIP and declined temporal precision to deficient speech perception (Fink et al., 2006; Vander Werff and Burns, 2011; Parbery-Clark et al., 2012), our results confirmed that training in TIP improved the phonetic identification measured by the VOT task. As stressed before, although the boundaries of categorical voiced distinctions in children with SLI were the same as in healthy peers, the correctness of performance in SLI was significantly lower (**Figures 5**, **6**). Only in EG after temporal intervention we observed significant improvement reflected in lowered β values (β_pre-test_ = 29.59 vs. β_post-test_ = 6.72). On the contrary, in CG after control intervention no significant differences were revealed (**Figure 7**).

Furthermore, it should be stressed that only following temporal intervention, the children with SLI reached the level of performance comparable to that observed in NC (non-significant differences between EG and NC). In contrast, following non-temporal intervention (in CG), the level of performance still remained below that of NC (significant differences between CG and NC, **Figure 8**).

Previous studies indicated beneficial effects of various interventions based on rapid auditory processing which resulted in amelioration of speech reception evidenced, e.g., by phonemic hearing in children with SLI (e.g., Tallal et al., 1996; Szelag et al., 2015). However, in the present study we confirmed that increased temporal precision in auditory processing resulted in more effective phonetic identification which seems to be more complex and nuanced than simple correct/incorrect phoneme differentiation measured with phonemic hearing tests.

In our previous studies, the effectiveness of intervention based on TIP was investigated in aphasic patients using the prototype version of *Dr. Neuronowski^®^* software (Szelag et al., 2014; Szymaszek et al., 2017). Patients were trained in sequencing two sounds presented in a rapid succession. It resulted in significant improvement of both TIP and speech reception (evidenced in phonemic hearing, global speech comprehension and VOT tests).

Referring to some theories on SLI, these children presented impaired working memory and selective attention which affected the phonological processes (Bishop et al., 1999; McArthur and Bishop, 2001; Vandewalle et al., 2012). Although, two intervention programs applied here (temporal vs. control) contained exercises focused on cognitive functions, like working memory, attention or executive functions, only the temporal intervention (addressed TIP) caused the improvement in speech perception, i.e., phonetic identification (studied here), as well as phonemic hearing and global speech comprehension (Szelag et al., 2015). Thus, one may emphasize that efficient temporal framework is fundamental for broader aspects of speech perception, but the training in working memory and attention incorporated in both applied interventions was not sufficient enough to improve speech perception skills in children with SLI.

For better understanding training-related benefits reported here in the context of our previous reports (Szelag et al., 2015), we conducted correlation analyses between phonetic identification indexed with β and phonemic hearing (indexed with the percent of errors) or TIP (TOT in millisecond time range).

### Correlations Between Phonetic Identification, Phonemic Hearing and TIP

Literature evidence indicated that some aspects of speech reception, i.e., both phonetic identification and phonemic hearing are rooted in millisecond temporal frame (Pöppel, 1997, 2009). In that context, we investigated whether the TOT values and the effectiveness of phonemic hearing tested in our previous study (Szelag et al., 2015) correlated with the β values obtained here, considered as the indicator of voiced contrast identification efficiency.

Only in EG in post-test both these measures (TOT and phonemic hearing) correlated significantly with the β values. Thus, the lower β (better contrast detection) was accompanied by better phonemic hearing and lower TOT (better TIP performance). It may suggest the existence of a neural mechanism underlying speech perception rooted in TIP which was improved during temporal intervention. The application of such exercises may result in a transfer of improvement from the trained non-verbal timing processing into the untrained verbal processing, i.e., some aspects of speech perception in which the temporal component is built-in. Hence, such transfer of improvement was documented in amelioration of both phonemic hearing and phonetic identification. One may hypothesize that following the temporal training the improved temporal acuity resulted in more coherent processing of both speech and non-speech stimuli. Such correlations in pre-test in both groups were non-significant probably due to more variable and less precise subjects’ responses. In CG the applied intervention did not influence TIP, thus, the preserved declined millisecond time frame resulted in non-significant relation ‘timing-speech perception.’

### Final Remarks

The obtained results revealed that children with SLI, despite lower correctness in phonetic identification, present the same as their healthy peers boundary location for categorical voicing contrast detection. Temporal intervention in children with SLI resulted in significant improvement of phonetic identification as compared to non-temporal control intervention. In CG intervention based on cognitive functions such as: working memory, attention, executive functions extended by typical speech therapy exercises (non-temporal control intervention) did not benefit speech perception assessed by phonetic identification.

## Author Contributions

AS: data acquisition, conducting therapy sessions, analysis and interpretation of data, and manuscript writing. AD: subject recruitment, data acquisition, conducting therapy sessions, analysis and interpretation of data, and contribution to manuscript writing. PU: analysis of psychometric function. ES: conceptualization and study design, analysis and interpretation of data, and manuscript writing. All the authors: final approval.

## Conflict of Interest Statement

ES and AS are the creators of the software package Dr. Neuronowski^®^, realized as a part of a project at the Nencki Institute with funding from the National Centre for Research and Development in Poland. The rights to the software lie with the Nencki Institute that has an agreement with Harpo Ltd., the company commercializing this software. ES and AS are not the owners of this technology nor do they have a direct financial arrangement with Harpo Ltd. The authors state that it does not affect the scientific validity of the results. The remaining authors state that the research was realized in the absence of any commercial or financial relationships that could generate any potential conflict of interest.

## Footnotes

1 VOT value of 0 ms reflects an overlapping of burst and vibration onsets.

2 In our preliminary data analyses, the sigmoid function seemed to be best fitted to data obtained in this study.

3 α and β exponents did not have a normal distribution.

4 Polish-speaking children perceived voiced category at VOT: –90, –80, –70, –60, –50, –40 ms, unvoiced at: –10, 0, +5, +10, +20 ms with the transition zone (a chance level performance) located for –30 and –20 ms. These categories were established on the basis of literature (Rojczyk, 2010) and our previous studies (Szelag and Szymaszek, 2014; Oron et al., 2015) as well as were confirmed in the present study (ANOVA, see below).

5 The data presented normal distribution.
